# P04-03 Does physical activity improve an interaction between motor control and cognitive functions in elderly?

**DOI:** 10.1093/eurpub/ckac095.057

**Published:** 2022-08-29

**Authors:** Vida Česnaitienė, Margarita Drozdova-Statkeviciene, Oron Levin, Marcin Zbigniew Ossowsky, Nerijus Masiulis

**Affiliations:** 1 Physical and social education department, Lithuanian Sports University, Kaunas, Lithuania; 2 Movement Control & Neuroplasticity Research Group, Deptartment of Kinesiology, KU Leuven, Leuven, Belgium; 3 Sport and recreation, Gdansk sports university, Gdansk, Poland; 4 Health and rehabilitation, Lithuanian Sports University, Kaunas, Lithuania

**Keywords:** Dual tasking

## Abstract

**Background:**

Normal aging is associated with progressive functional loss in many cognitive domains, including working memory, attention (van Raalten et al., 2008) and executive functions (Nyberg et al., 2008), responsible for the control of behavioral activities (Miller & Cohen, 2001). Research aim was to evaluate postural control and executive function during dual tasking in physically active and inactive old adults.

**Methods:**

Participants were 42 older healthy human males and females (Mean age: 70.17±6.08 years). Posturography method with a single piezoelectric force plate was used to measure postural sway activity. For the evaluation of cognitive functions, we used Word Memory task with ten audio-recorded words (Lithuanian nouns) in each trial, and the Mathematical Processing Task, where negative or positive one-digit integer-numbers (10 in total) were presented in each trial at 2-second intervals. Physical activity of participants was evaluated according to WHO recommendations.

**Results:**

The study showed that there was a strong correlation between physically active time spent and balance behavior. The balance of physically active older people was statistically significantly more stable when they performed cognitive tasks than that of those who were physically inactive. Dual-task interferences on postural sway were evident in both Word Memory task and the Mathematical Processing Task conditions. Dual-task effect on Mathematical Processing Task and Word Memory task was not statistically different.

**Discussion:**

Taken together, we suggest that physical activity improves proprioceptive control which also improves balance control. In dual tasking, more attention is required in cognitive tasking, so better proprioception allows for better balance control with fewer attention resources. However, it is also evident that participants can reduce sway activity and increase balance stability by increasing attentional control.

